# Gut Microbiome Composition and Metabolic Status Are Differently Affected by Early Exposure to Unhealthy Diets in a Rat Model

**DOI:** 10.3390/nu13093236

**Published:** 2021-09-17

**Authors:** Paolo De Marco, Ana C. Henriques, Rui Azevedo, Susana I. Sá, Armando Cardoso, Bruno Fonseca, Joana Barbosa, Sandra Leal

**Affiliations:** 1 TOXRUN—Toxicology Research Unit, University Institute of Health Sciences, CESPU, CRL, 4585-116 Gandra, Portugal; paolo.demarco@iucs.cespu.pt (P.D.M.); ana.henriques@cespu.pt (A.C.H.); rui.azevedo@iucs.cespu.pt (R.A.); joana.barbosa@iucs.cespu.pt (J.B.); 2 Center for Health Technology and Services Research (CINTESIS), Rua Dr. Plácido da Costa, 4200-450 Porto, Portugal; sasusana@med.up.pt (S.I.S.); cardosoa@med.up.pt (A.C.); 3 Department of Biomedicine—Anatomy Unit, Faculty of Medicine, University of Porto, Alameda Prof. Hernâni Monteiro, 4200-319 Porto, Portugal; 4 UCIBIO—Applied Molecular Biosciences Unit, Biochemistry Laboratory, Biological Sciences Department, Faculty of Pharmacy, University of Porto, Rua Jorge de Viterbo Ferreira 228, 4050-313 Porto, Portugal; brunofonseca@ff.up.pt; 5 UCIBIO, REQUIMTE, Toxicology Laboratory, Biological Sciences Department, Faculty of Pharmacy, University of Porto, Rua Jorge de Viterbo Ferreira 228, 4050-313 Porto, Portugal

**Keywords:** high-sugar diet, cafeteria diet, microbiota, metabolic dysregulation, young animal

## Abstract

Childhood is a critical stage of development during which diet can have profound influence on the microbiota–host interactions, leading to potentially lifelong impacts. This study aimed to investigate whether the consumption of cafeteria diet (CAFD) and sugary drinks during early rat life alters the structure of the gut microbial community and the metabolic activity. Four-week-old male Wistar rats (*n* = 27) were fed a standard chow diet with ad libitum access to water (CD) or to sucrose solution (HSD), and a third group was fed with CAFD and a sucrose solution for 14 weeks. HSD and CAFD consumption induced alterations in Firmicutes to Bacteroidetes ratio, Proteobacteria, and Verrucomicrobia. HSD increased the abundance of *Barnesiella*, whereas CAFD induced a depletion of Saccharibacteria. CAFD increased total white adipose tissue (WAT) weight (*p* < 0.0005) compared to CD. When CAFD was compared to HSD, a significant difference was found only for retroperitoneal WAT (*p* < 0.0005). Unhealthy diet-fed groups presented higher glucose (*p* < 0.0005), total cholesterol and creatinine serum levels (*p* < 0.005) compared to the CD rats. Early-life consumption of HSD, and of CAFD even more so, can have long-lasting negative effects on metabolic function. The gut microbiota communities were distinctively perturbed by diet composition.

## 1. Introduction

The gut microbiota acts as a dynamic organ, a key regulator of host physiology, which can be a critical determinant in health and disease [1,2,3]. A large body of evidence has demonstrated the capacity of gut microbiota to extract nutrients from the diet, affecting energy harvest and energy storage in the host [4,5,6,7]. In addition, the microbiota plays a major role in priming and regulating intestinal barrier function and host immunity [2,8]. It has been shown that microbiota–host interactions are initiated at birth and, until a stable microbial community is established, dietary patterns are indicated as strong drivers of microbiota composition [9,10], and of metabolic and immune homeostasis [11]. Moreover, the disruption of the gut microbiota toward a proinflammatory profile in early life emerges as a potential mechanism for the initiation and/or persistence of pathological states [2,12]. Therefore, dietary influence on the gut microbiota during early-life developmental stages deserve to be further investigated.

The inflammatory impacts of an altered microbiota have been revealed well beyond the gut, such as on brain function and behavior [13]. The hypothesis that early-life diet plays a key role in programming how the microbiota–gut–brain axis may influence many biobehavioral aspects and stress responses is an emerging concept [13,14]. Although several studies suggest that short and long-exposure to a cafeteria diet induces memory impairment associated with a worse metabolic profile, regarding the effects induced by a high-sugar diet the results are inconsistent. In addition, the underlying mechanisms responsible for memory deficits and neuroinflammation associated with high sugar intake remain unclear. Nevertheless, excessive sugar consumption has been associated with dysbiosis and brain impairment, namely hippocampal memory function [14], suggesting that early exposure to added sugar might affect the microbiota–gut–brain communication.

There is strong evidence that consumption of high-fat and high-sugar diets shifts gut microbiota communities, affecting metabolic endotoxemia and the onset of obesity. Increased energy extraction from food, intestinal permeability and systemic inflammation, as well as deregulation of gut hormone secretion, are all potential mechanisms through which the gut microbiota influences host metabolism [4,5,6,8]. Nevertheless, impairment of glucose tolerance induced by high-fat and sugar liquid diets may be associated with increasing levels of *Escherichia coli* [15], indicating that dietary habits can cause a shift in the bacteria communities in the gut.

The gut microbiota in both rats and humans is mainly composed of Bacteroidetes and Firmicutes, with minor percentages of Proteobacteria, Spirochaetes and Cyanobacteria in rats or Actinobacteria, Proteobacteria and Verrucomicrobia in humans [6,16,17]. However, shifts in the microbial community structure influenced by food composition, particularly macronutrients, are known to occur [1,12,15,18,19]. In rat microbiota, high-fat diets seem to enrich phylum Bacteroidetes [19] and complex-carbohydrate diets promote the proliferation of genus Prevotella [20]. It is becoming evident that diet-induced changes in populations of gut microbiota can play a role in the development of obesity and related disorders [15,19]. Diet-induced obesity has been successfully demonstrated in animal models, using typical high-fat purified diets [21,22,23] or cafeteria diet, composed of a variety of highly palatable and caloric foods [24,25,26,27,28]. The cafeteria diet model, reflecting more accurately human dietary patterns, is a useful model to study metabolic dysfunction [25,26].

Experimental studies using high-fat diets in rodents have shown a decrease in the Firmicutes to Bacteroidetes ratio, loss of microbial diversity and increased pro-inflammatory potential [19,21,24,25], including high levels of lipopolysaccharide (LPS) and activation of Toll-like receptor (TLR)-4 and TLR-2 [26]. Therefore, early-life diet can interfere with the time frame of microbial colonization and shape the infant gut microbiota, with the resulting effects ranging from transient to long-lasting. Shifts in microbial community composition often require persistent dietary selective pressure to become permanent and, even so, the durability of these changes remains uncertain [18].

All evidence indicates that infancy and early childhood are stages of greater vulnerability to the effects of dietary choices that can profoundly shape gut microbiota, increasing the risk for metabolic, cognitive and behavioral disorders in adulthood. Although there is evidence of the effects of cafeteria and high-sugar diets on adult animals’ metabolic dysfunction, less is known about the effects of early exposure to these two different obesogenic diets on the shifting of gut microbial composition, particularly how they affect metabolic capacities and whether these are related with specific changes in gut microbiota. Therefore, the characterization of the changes induced in gut microbiota diversity by distinct unhealthy dietary patterns that can contribute to early metabolic impairment are essential to better understand the impact of the gut microbiota on health and disease.

## 2. Material and Methods

### 2.1. Animals

Male Wistar rats (*n* = 27) were obtained from the colony of the Institute for Molecular and Cell Biology/i3S (Porto, Portugal). They were maintained in the animal facility of the Department of Biomedicine of the Faculty of Medicine, University of Porto and allowed to acclimatize. Animals were kept under standard laboratory conditions (22 ± 1 °C and a 12 h light/dark cycle) with free access to water and standard rat chow (4RF21/C Mucedola, Milan, Italy). Before dietary intervention, rats were housed two per cage to avoid social isolation. All efforts were made to minimize the number of animals used, animal discomfort and stress.

### 2.2. Dietary Treatments

At 4 weeks of age, rats were weighed and distributed into three dietary treatment groups (*n* = 9 per group) for 14 weeks. Composition of the dietary intervention is detailed in Supplementary Table S1. The chow diet (CD) group, which will be also referred to as the “healthy diet” group, was fed with the standard chow and had free access to water. The high-sugar diet (HSD) group was fed with the standard chow and had free access to 30% sucrose solution. The cafeteria diet (CAFD) group was fed with the standard chow mixed with a selection of palatable foods and had free access to 15% sucrose solution. The HSD and CAFD are also referred to as “unhealthy diets”.

### 2.3. Biometric Measurements

Food and liquids were available ad libitum, daily replaced and quantified. Food, water and sucrose solution intakes were calculated by subtracting the amount left in the cage/bottle from the total amount of food and solution provided. The individual consumptions were calculated by dividing the total food and liquid intake from each cage by the number of rats. Energy intake was calculated from the diet macronutrient composition as estimation of metabolizable energy based on the Atwater factors, assigning 4, 4 and 9 Kcal/g for the available carbohydrate, protein and fat, respectively.

Body weight (BW) was monitored weekly throughout the entire experimental period and body weight gain per week was calculated.

The Lee obesity index was assessed at the beginning and at the end of the study, and was calculated as described previously [27]. Energy efficiency was also assessed for each treatment weekly by dividing the body weight gain by the total calories (Kcal) consumed [28].

### 2.4. Adiposity Measurements and Biochemical Analysis

At the end of the experimental period, rats were fasted for 12 h and euthanasia was performed under deep anesthesia induced with sevoflurane (SevoFlo, Abbott Laboratories Ltd., Maidenhead, UK). The perigonadal, retroperitoneal and abdominal white adipose tissues (WAT) were dissected from each rat and weighed before being flash frozen in liquid nitrogen and stored at −80 °C. WAT weights were further used to calculate the total WAT weight; all values are expressed in grams (g). The relative WAT was determined as percentage of body weight.

Blood was drawn from the left ventricle into BD Vacutainer^®^ tubes and centrifuged at 6000 rpm for 10 min. Serum aliquots were frozen at −80 °C until further analysis. Concentrations of glucose, triglyceride (TG), total cholesterol, high-density lipoprotein cholesterol (HDL-c), low-density lipoprotein cholesterol (LDL-c), creatinine and urea were measured using the Prestige 24i automated analyzer (Cormay, Tokyo Boeki, Japan), following the manufacturer instructions. All reagents were supplied by Cormay PZ (Warsaw, Poland).

### 2.5. Fecal Sample Processing

Good-quality fecal samples were obtained from all HSD and CAFD animals; samples from 4 CD animals were not stored properly and had to be discarded, leaving samples from just 5 of the 9 CD animals. Samples were collected into sterile tubes, immediately frozen in liquid nitrogen and stored at −80 °C until processing. Genomic DNA was extracted from samples (ca. 0.180 g) using QIAamp PowerFecal DNA Kit (QIAGEN). DNA was quantified in a Nanodrop™ 2000 spectrophotometer (Thermo Scientific, Waltham, MA, USA).

### 2.6. 16S Ribosomal RNA Gene Sequencing

Next-generation sequencing was performed at Molecular Research LP (Shallowater, Texas, USA) using a reengineered version of a bTEFAP^®^ method [29,30], adjusted to the Illumina MiSeq platform. Briefly, the V4 variable region of the 16S rRNA gene was amplified using primers 515F GTGCCAGCMGCCGCGGTAA and 806R GGACTACHVGGGTWTCTAAT, with barcodes on the forward primer. A single-step 30 cycle PCR was carried out, using the HotStarTaq Plus Master Mix Kit (Qiagen, USA), under the following conditions: 94 °C for 3 min; 28 cycles of 94 °C for 30 s, 53 °C for 40 s and 72 °C for 1 min; and a final elongation step at 72 °C for 5 min. After amplification, PCR products were checked in a 2% agarose gel and samples were equivalently pooled, according to DNA concentration. Samples were then purified with Ampure XP beads (Agencourt Bioscience Corporation, MA, USA). Sequencing was performed using the Illumina MiSeq chemistry per manufacturer’s procedures. Raw reads were submitted to the European Nucleotide Archive (ENA-EBI) under project identifier PRJEB23897 (sample accession numbers ERS2045493 to ERS2045515).

The resulting sequence reads were analyzed with the Quantitative Insights into Microbial Ecology (QIIME) software (v1.9.1) [31]. Briefly, reads were joined, stripped of barcodes, checked for quality (quality score > 25), and demultiplexed. Length filtering was performed using the SeqKit toolkit [32]. Sequences were prefiltered by removing those that did not hit operational taxonomical units (OTUs) in the reference database with at least 60% identity; after this filter, they were then clustered into OTUs with at least 97% identity through an open reference approach, using the UCLUST algorithm. Taxonomy was assigned using SILVA database release 128 (Supplementary Figure S1).

Alpha and beta diversities were analyzed within QIIME. The Shannon index (diversity) and the Chao1 index (richness) were calculated with rarefied data (227762 sequences per sample). Principal coordinate analysis (PCoA) plots were constructed based on weighted Unifrac distances and visualized using EMPeror v0.9.51-dev [33], and beta-diversity significance was assessed by ANOSIM (Analysis of similarities).

Linear discriminant analysis (LDA) effect size (LEfSe) testing [34] was performed pairwise (between groups) in order to identify differentially abundant bacterial taxa (from phylum to species level). The differences were considered to be significant at LDA scores > 3.0 and *p* < 0.05. Associations between metadata and microbial community abundance or function were performed with MaAsLin [35] with a minimum taxon relative abundance of 0.0001, a minimum taxon prevalence of 0.01 and a significance threshold of 0.025.

### 2.7. Statistical Analysis

Data are expressed as the mean ± standard deviation (SD). One-way ANOVA was used to determine whether there were group differences in body weight, weight gain, WAT weights, energy intake and efficiency. To compare the differences between two groups, *t*-test was used and, when *p* < 0.05, differences were considered statistically significant.

## 3. Results

### 3.1. Unhealthy Diets Differently Affect Body Composition and Metabolic Phenotype

**Figure 1:**
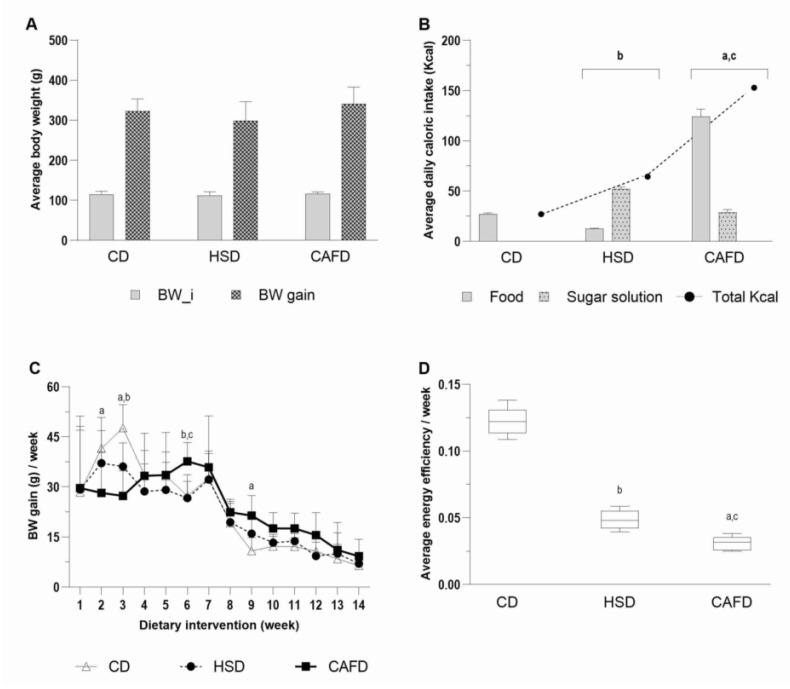
Graphical representation of the effects of the diets on energy intake and body composition parameters. (**A**) Body weight (g) at the beginning (BW_i) and weight gain after 14 weeks of diet intervention. (**B**) Total daily energy (Kcal) and caloric intake in food and sugar solution diets. (**C**) Body weight variation across the entire dietary experiment. (**D**) Average energy efficiency per week. BW, body weight; CD, chow diet; CAFD, cafeteria diet; HSD, high-sugar diet. Values represent mean ± SD (*n* = 9). Common letters indicate statistically significant differences between the associated groups: ^a^ CD vs. CAFD; ^b^ CD vs. HSD; ^c^ HSD vs. CAFD.

Average body weight variation and food intakes are shown in Figure 1. The mean body weights of the groups were similar at the beginning and there was no significant diet effect on the total body weight gain (Figure 1A). However, body weight gain was variable over the experimental period and between diet-treated groups with variable contents of macronutrients and added sugar intake (Supplementary Table S1). The average daily caloric intake from solid food, drink and total energy intake were significantly higher in HSD or CAFD rats (*p* < 0.0001) (Figure 1B). CAFD-fed rats consumed two and five times more calories than those consumed by HSD and CD animals, respectively. Despite the higher total caloric intake of HSD rats compared with CD group (Figure 1B), energy intake from solid chow food reduced (*p* < 0.0001) and sucrose solution was the source of extra energy. Total body weight gain of HSD group tended to be lower than in the other groups (Figure 1A). During the 14 weeks of dietary intervention, CD group showed a higher weight gain at the second and third weeks (Figure 1C). In these two weeks, CD were heavier than CAFD-fed rats (*p* < 0.005; *p* < 0.0001, respectively) and only in the third week did they exhibit more weight gain than HSD rats (*p* < 0.05). At the sixth week of diet intervention, the CAFD rats exhibited significant weight gain (*p* < 0.05) when compared with the other groups. From that week on, CAFD group maintained the highest weight gains; however, difference was statistically significant only in the ninth and eleventh weeks when compared to CD group (*p* < 0.05). During the second and third weeks, the rats in the HSD group gained more weight than those in the CAFD group, a tenuous transitory tendency (Figure 1C). The average of energy efficiency per week was higher in the CD group (Figure 1D), compared to both HSD and CAFD (*p* < 0.0001). Between the unhealthy diets, energy efficiency was higher in HSD animals (*p* = 0.0002).

The Lee obesity index was comparable among groups fed with chow, HSD and CAFD both at the beginning (306 ± 11, 307 ± 14 and 310 ± 20, respectively) and at the end of the study (304 ± 13, 296 ± 20 and 314 ± 24, respectively). The upper and lower limits of the 95% confidence intervals (CI) for the Lee obesity index showed higher variations in rats fed with HSD (95% CI = 280, 312) and CAFD (95% CI = 294, 334) than for CD rats (95% CI = 293, 315). Animals fed the CAFD presented the highest absolute WAT (perigonadal, retroperitoneal and total) weights and percentage of total WAT relative to body weight (Table 1), which were significantly higher when compared with animals in CD group. HSD-fed rats showed a tendency to increase body fat mass compared with CD group. A significant difference between unhealthy diets on WAT was found only for retroperitoneal WAT weight with the higher increase displayed by CAFD-fed rats.

Serum biochemical parameters are shown in Table 1. Total cholesterol levels were significantly increased in both HSD and CAFD rats compared to values of the CD group. Moreover, an increasing trend in LDL-c levels was seen in both HSD and CAFD groups. Serum TG levels and TG/HDL-c ratio were significantly increased in CAFD rats comparing with the CD group, but not compared with HSD rats. Glucose levels were higher in HSD and CAFD rats than in the CD group, but no difference was observed between the two unhealthy diets. HSD and CAFD also caused a significant increase in creatinine, while urea levels were increased only in CAFD group.

### 3.2. Taxonomic Classification of 16S rRNA Sequence Reads

QIIME was used to process the reads and cluster the sequences into operational taxonomic units (OTUs) using the SILVA database as a reference. General sequencing statistics are reported in Table 2, while detailed sample statistics can be found in Supplementary Table S2.

### 3.3. Distinct Effects of Unhealthy Diets on Gut Microbiota Composition

The gut microbial communities of the 23 rats were analyzed by sequencing the V3-V4 regions of the 16S rRNA gene.

Phylogenetically, the average gut microbiota taxonomical composition of the CD group was: Firmicutes (74.4%), Bacteroidetes (21%), Verrucomicrobia (2%), Tenericutes (1.7%), Proteobacteria (0.23%) and other phyla (0.7%). The vast majority (on average 93.7%) of the phylum Firmicutes belonged to the Clostridia class.

**Figure 2:**
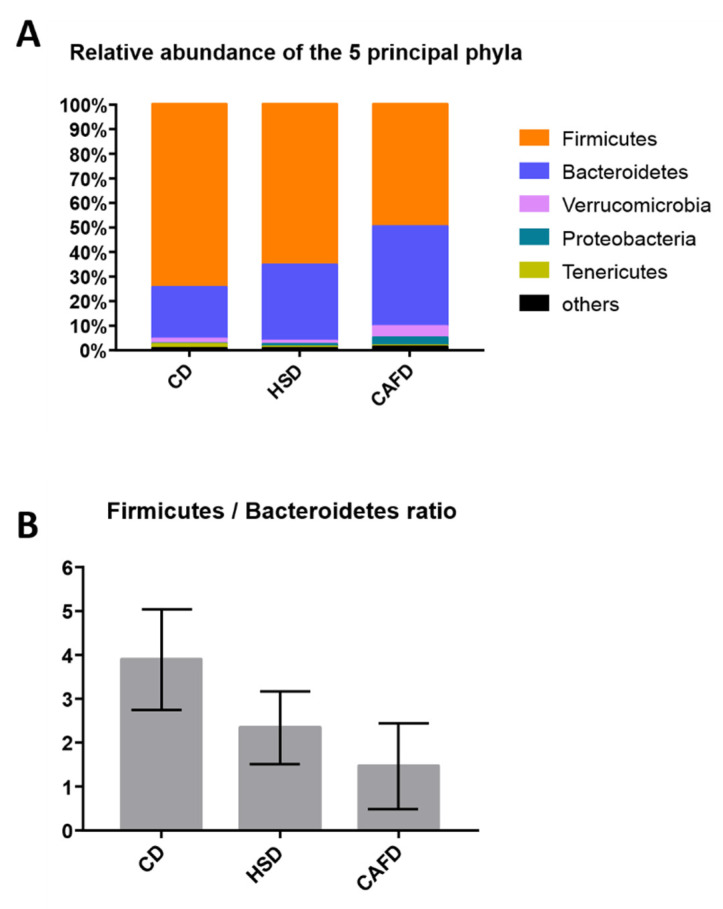
Graphical representation of the microbial community composition. (**A**) Major bacterial phyla in the gut microbiota of rats after 14 weeks of chow diet (CD), high-sugar (HSD) or cafeteria diet (CAFD) feeding. Bars represent the average relative abundance of each phylum in the 3 different diet groups. Each phylum is represented by a different color (*n* = 5 to 9 rats per group). (**B**) Firmicutes to Bacteroidetes ratio among groups. Values are expressed as mean  ±  SD (*n* = 5 to 9 rats per group).

In general, rats fed with unhealthy diets (HSD or CAFD) were associated with a reduction of Firmicutes and Tenericutes and an expansion of Bacteroidetes and Proteobacteria (Figure 2A and Supplementary Table S3). The Firmicutes to Bacteroidetes ratio was significantly decreased in CAFD animals (*p* < 0.01), while the reduction seen in HSD was not statistically significant (Figure 2B).

**Figure 3:**
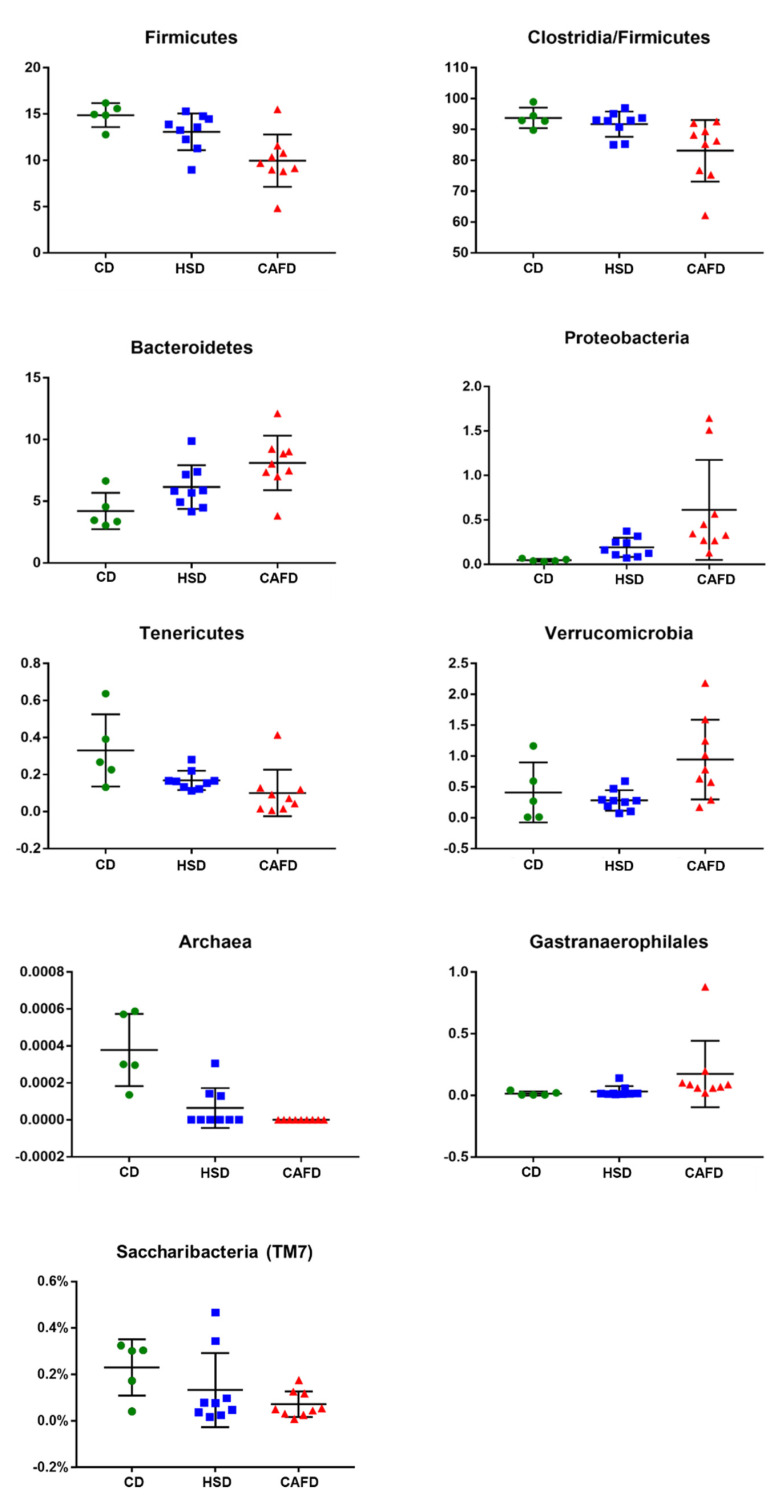
Graphical representation of the effects of different diets on taxa frequencies of the most representative taxa in samples collected from male Wistar rats fed with chow diet (CD) (*n* = 5), high-sugar (HSD) (*n* = 9) or cafeteria diet (CAFD) (*n* = 9) for 14 weeks. Values are expressed in percentage and represent mean ± SD.

Compared to the CD group, phylum Verrucomicrobia (composed almost exclusively by genus *Akkermansia*) was more abundant in the CAFD animals and less abundant in the HSD animals (Figure 3). Class Clostridia of the Firmicutes was less prevalent in the HSD rats and even less so in the CAFD animals when compared with CD group. Phylum Saccharibacteria (formerly “group TM7”) was also significantly depleted in CAFD animals compared to controls (CD).

Another general trend in the distal-gut microbiotas of the animals subjected to the two unhealthy diets was the loss of Archaea: although these methanogenic Euryarchaeota were already rare in all CD rats (average 0.002%), this phylum was drastically depleted by the two other dietary regimens.

Class Mollicutes was less abundant in animals fed the HS diet compared to CD, and even more depleted in CAFD rats, and many other significant differences were detected at the level of single genera (as detailed in Supplementary Table S3).

**Figure 4:**
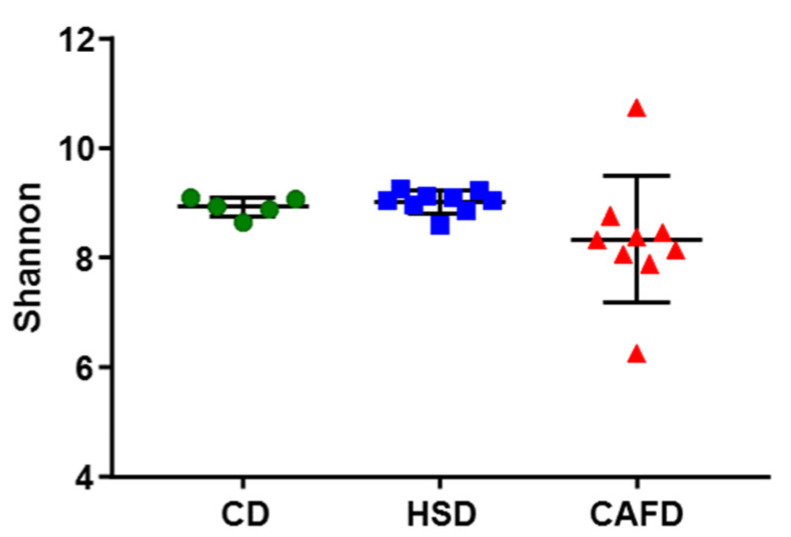
Comparisons of Shannon diversity values.

**Figure 5:**
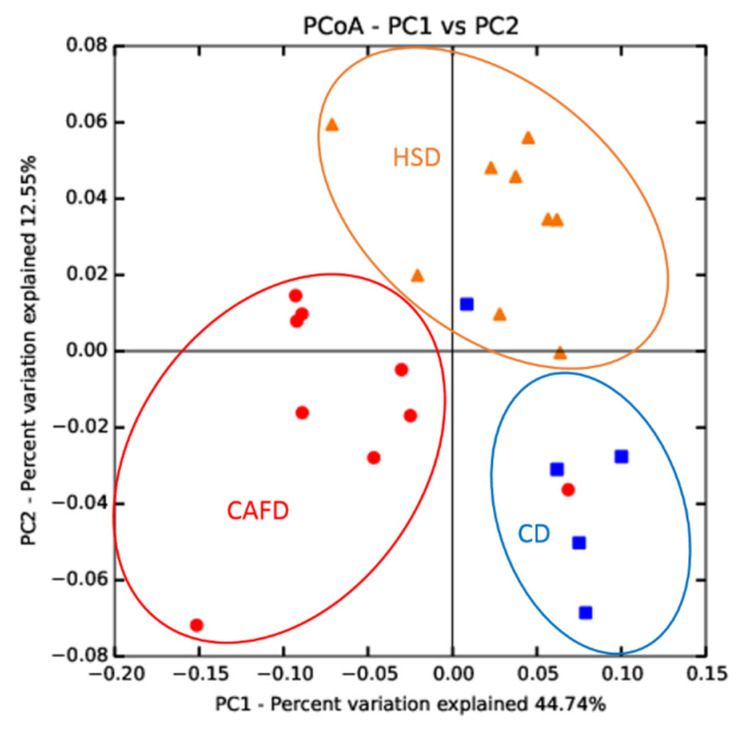
Graphical representation of the principal Component Analysis comparison of the microbiota in the 23 male Wistar rats.

Despite the extensive differences in the prokaryotic phylogenetic composition of the 3 groups of samples, the overall alpha-diversity indexes did not show significant variations. Figure 4 shows the results in the Shannon diversity index H, and other alpha-diversity indexes presented similar pictures. The HSD did not lead to significant alterations in diversity compared to the CD. The CAFD caused an evident increase in internal sample dispersion, with an overall decrease in average diversity, but this decrease was not significant in the direct comparison with the other two sets of samples. However, if markedly outlying samples (CAFD3 and CAFD8) are omitted from the analysis, average alpha-diversity becomes highly significantly (*p* < 0.001) lower in CAFD than in the other two groups. Beta-diversity analysis by weighted UniFrac distances also showed significant (*p* = 0.001) differences between the three groups of samples (Figure 5).

## 4. Discussion

It is recognized that both human and rat gut microbial communities undergo a process of development along with their hosts [2,12,17,18,24]. In the developmental processes of gut microbiotas, early ages are considered unique transitional stages, owing to their distinct composition and peculiar gut functional features compared with the adult [2,12]. Moreover, the core microbial composition can be shaped by early dietary patterns, which are pointed out as major developmental determinants [10,21,36]. Eating patterns characterized by low nutritional variety, caloric foods and added sugar are related to the impoverishment of gut microbiota [19,21,23,37]. Our results in young rats support and extend this concept, by showing that the two unhealthy diets studied clearly influenced the composition and diversity of gut microbiota, such as the Firmicutes to Bacteroidetes ratio, the disappearance of methanogenic Archaea, the fluctuating abundance of the *Akkermansia* genus, the loss of Mollicutes, and the changes in other minor taxa. In addition, the early consumption of both CAFD and HSD induced metabolic dysfunction and some renal function impairment. The diet-related effects showed that the combination of fat and sugar can have more severe effects on metabolism than sugar alone, corroborating previous studies that suggest the involvement of independent mechanisms [15]. Our results provide support to the notion that early-life consumption of unhealthy diets can disrupt microbial communities [3,38,39], leading to long-lasting consequences for health outcomes [3,13].

Concerning body weight gain and/or fat accumulation, diverging conclusions were proposed for rodents fed unbalanced diets with excess of lipids, sugar or both combined [15,19,25,26,27,40]. Chronic exposure to a highly saturated fat diet induced an increase in body weight gain and visceral fat accumulation in both Wistar rats and C57BL/6J mice [36]. When comparing CAFD with a high-fat diet, higher body weight gain and adiposity in CAFD-fed rats were associated with a distinct metabolic and inflammatory phenotype [41,42]. Nevertheless, 10 week-old Wistar rats exposed to a CAFD showed no effect in body weight gain and WAT accumulation [19], while 3-week old rats fed a CAFD displayed an increase in both parameters [43], suggesting age-specific effects of diet on body adiposity. Herein we showed that 4 week-old Wistar rats fed a CAFD for 14 weeks did not gain more body weight than rats fed a HSD or a chow diet, but it did induce a significant increase of visceral adiposity. The higher body weight gain reported previously [43] may be explained by the different food products included in the CAFD mix, but probably also by a strong effect of housing animals individually at early age [44], while social isolation was avoided in the present study. Genetic factors are clearly also at play, since dietary interventions can promote larger body weight gains in some obesity-prone rodent strains, whereas other breeds are resistant [44,45]. Indeed, our young rats fed a CAFD for a long term displayed a greater variance in Lee obesity index despite no significantly higher average value, coupled with the highest visceral WAT weight. These data indicate that the anthropometric parameters such as the Lee obesity index and body weight gain were insufficient to discriminate the WAT accumulation and its distribution. Our results, by analogy, hint that higher consumption of palatable energy-dense foods and soft drinks by young children [46,47,48] may contribute to an early onset of metabolic dysfunction, which can progress to greater metabolic disorders in adulthood [3,12,49].

Adverse metabolic effects due to the consumption of sugary beverages have been reported both in animals and human studies [22,39,48,50], including hyperglycemia, insulin resistance, dyslipidemia and hyperleptinemia [45,50], which can occur independently of a significant increase in body weight [39,51]. Corroborating and extending these previous reports, we observed that the early consumption of a HSD had no effects on Lee obesity index and body weight gain, but induced a non-significant trend toward increased visceral adiposity compared to rats fed the standard chow diet. In addition, HSD rats showed a significant increase of blood glucose and total cholesterol levels compared to CD rats. We also observed a tendency towards elevated serum TG levels in HSD-fed rats, which may be due to hepatic de novo lipogenesis from fructose [15]. Comparing the metabolic outcomes in HSD and CAFD, the only significant difference that we observed was an increase in the retroperitoneal WAT weight in CAFD-fed rats. Other subtler differences were found in the general metabolic profile, which showed a tendency to be worse in the rats that consumed CAFD. These observations are consistent with the possible dysregulation of different metabolic pathways [15] supporting the view that the combination of added sugar and saturated fats can be especially detrimental for metabolic function and inflammation [40,42], potentially through its influence on gut microbiota composition.

Dietary fiber, along with its beneficial influence on gut microbiota [1,52], seems to have an important role in gut-derived uremic toxins, yielding lower serum urea and creatinine levels and preserving renal function [52]. Serum creatinine and urea are biochemical parameters used to evaluate the damage of kidney function and recently they have emerged as potential metabolic markers of the communication between gut and kidney [52]. Our data show that CAFD induced an increase in both urea and creatinine serum levels, while HSD increased just creatinine, indicating some renal function impairment. In agreement with previous reports [15,52], our results suggest that both unhealthy diets induce the expansion of bacterial groups (such as the Enterobacteriales) that may generate uremic toxins. Moreover, a decrease of dietary fiber consumption can be a factor limiting microbiota diversity, affecting the production of short-chain fatty acids (SCFAs), leading to gut barrier dysfunction [1,8,21]. Therefore, dysbiosis induced by diet can be contributing to the accumulation of uremic toxins [15], which can, in turn, trigger local and systemic chronic inflammatory mechanisms, favoring the progression of renal damage coupled with metabolic disorders [52].

A strong body of evidence supports the notion that gut microbiota affects energy harvesting from the diet and energy storage, which may lead to an increase of body fat content in the host [4,5,6,7,53]. Most of this knowledge has come from studying germ-free animals. Indeed, gut colonization of adult germ-free C57BL/6J mice with microbiota from conventionally raised obese mice [5] or even from normal donor mice [7] resulted in a greater gain of visceral body fat compared to the germ-free controls, despite lower diet chow intake [5,7]. Moreover, altered gut microbiota induced by a diet rich in unsaturated fats seems to offer protection from lard-induced weight gain [26]. Therefore, it was proposed that the higher body fat content may not be caused just by the amount of calorie intake, but rather by the increased extraction of energy from food by the gut microbiota [7]. Previous findings linked gut microbiota with diet-induced obesity and inflammation [26,54], leading to distinct outcomes depending on the dietary pattern [15]. Accordingly, we observed that HSD and CAFD distinctly affected the gut microbiota composition, suggesting that early perturbations in the assembly of the gut microbial community may differently affect the mechanisms underlying metabolic dysregulation. Structural and functional alterations of gut microbiota by unhealthy diet was shown to worsen metabolic dysfunction via an increase in systemic inflammation, but also through alterations in the neuroendocrine circuits that regulate the ingestive behavior and energy metabolism [53].

The interplay between unhealthy diets and gut microbiota has been investigated in several animal models and the major negative changes were often attributed to the diet fat content [25,55]. Changes in the Firmicutes to Bacteroidetes ratio have been repeatedly reported to be associated with the so-called high-fat diets, including lard, Western diet and CAFD [19,21,26]. The alteration in the proportion of Firmicutes and Bacteroidetes phyla with regard to high-fat diets seems to be dependent on the type of fat content [19,26,56]. Diets rich in both saturated and unsaturated fat were shown to induce a decrease of Firmicutes and an increase in Bacteroidetes in mice, compared to mice fed a low-fat diet [56]. In this study, feeding young rats for 14 weeks with a saturated fat-rich diet (CAFD) induced a decrease in the Firmicutes to Bacteroidetes ratio and increased the abundance of Proteobacteria, results that are consistent with prior reports [19,25,26,55]. In addition, our data revealed that early HSD feeding induced a reduction of Firmicutes and Mollicutes, while increasing the abundance of Bacteroidetes and Proteobacteria. Changes in the abundance of Proteobacteria was reported in children who consumed a calorie-dense diet with low fiber content [10], suggesting that the Proteobacteria may function as a diet-sensitive phylum, which signals a state of gut dysbiosis in the host [57]. Phylum Verrucomicrobia (practically exclusively genus *Akkermansia*) was less abundant in HSD than in the controls. In an apparent contradiction, CAFD-fed rats gained more visceral WAT weight, showed worse biochemical parameters, but presented an increase in the abundance of *Akkermansia*, suggesting that the competition between bacteria for substrates may lead to an inability to protect from bacterial pathogens [58]. Supporting this concept, it has been demonstrated that changes in gut microbiota induced by chronic dietary fiber deficiency, including an increased abundance of *Akkermansia*, can induce a switch in metabolism of gut microbiota species from fiber degradation to mucus glycan degradation [59]. Although both unhealthy dietary patterns changed the gut microbiota composition compared with standard chow-fed rats, some subtler differences were evident between the two treatments, such as a reduction of classes Clostridia and Mollicutes, especially pronounced in CAFD-fed animals. Compared with CD, CAFD showed a clear tendency towards a reduced richness of the microbiota, albeit accompanied by an increased heterogeneity between animals, which confounds the significance in the direct comparison. These results are aligned with the concept that unhealthy dietary patterns in early-life have a deleterious effect on the development of gut microbiota, shifting toward a more unwholesome composition [1,3,10,39,55].

Early in the development of gut microbiota, compositional feedback loops (positive or negative) may have a strong role in de/stabilizing the microbiota during that critical time of adaptation [18]. Therefore, early exposure to high-energy foods may trigger unusual feedbacks in the gut environment, which may induce an anomalous directional change in the microbial community. Indeed, derailment of the gut microbiota developmental process caused by early exposure to unbalanced diets was demonstrated in C57BL/6 mice fed a commercial high-fat diet [55] and in Sprague Dawley rats provided with a sugary solution [14,39]. Although exposure to dietary patterns in early life can be particularly important for the development of obesity and metabolic disorders [2,12], there are few animal studies exploring how early-age microbiota perturbation leads to lifelong consequences [14,39,55]. It was demonstrated that a sugary liquid diet in juvenile male Sprague-Dawley rats resulted in an increase in Proteobacteria and Bifidobacteriales and a reduction of Prevotellaceae and Ruminococcaceae [39]. These shifts in gut bacteria were also associated with impaired glucose tolerance independent of obesity [14,39] and negatively correlated with memory performance [14]. Moreover, in young adult Wistar-Kyoto rats, high-fat and high-fructose diets induce an increase of plasmatic LPS and pro-inflammatory mediators brought about by an increase in Bacteroidetes and Proteobacteria [15]. Other specific bacterial groups, such as *Clostridium* and *Escherichia*, may contribute to systemic inflammation through the secretion of enterotoxins or the diffusion of LPS through the intestinal barrier [20]. In addition, it was shown that food emulsifiers increased pro-inflammatory mediators, inducing colitis and metabolic syndrome in mice [60], suggesting that emulsifiers present in the processed foods of CAFD might exert additional detrimental effects on the gut microbiota.

The gut microbiota may affect host metabolism by changing the hormonal milieu and the immune response [8,13,21]. When occurring at a young age, this may strongly influence growth and development. Beyond performing as an energy source to the host, SCFAs produced by gut microbiota act as signaling molecules, regulating immune cells through TLR4 [8,13,21] and also interplaying with neuroendocrine mechanisms that regulate food intake and energy [45]. Indeed, SCFAs can promote the release of the endogenous peptides glucagon-like peptide-1, peptide YY and ghrelin, confirming the link between SCFAs, appetite and energy homeostasis [8,45]. The influence of an unwholesome gut microbiota is not limited to the dysregulation of metabolic pathways and immune responses of peripheral organs [3,11,13,36,61], as it also affects brain development and function [1,13,14]. It was demonstrated in rats that early chronic sugar intake can lead to long-lasting memory impairment [14], while CAFD-induced memory impairment was accompanied by anxiety-related behaviors [51]. Therefore, early disturbance of the gut-brain communication by an unhealthy diet can affect the host brain development, which might aggravate metabolic dysfunction, but also contribute to cognitive and behavioral disorders.

## 5. Conclusions

Our results show that the development of the gut microbiota community can be affected by early exposure to unwholesome nutritional regimens, leading to distinct compositional features in the adult gut, with depletion of particular microbial classes or families. In addition, we demonstrate that consumption of CAFD and a sugary beverage entailed long-lasting negative metabolic effects and some renal impairment, probably influenced by distinct effects on gut microbiota composition. Since gut microbes exert their activities collectively and through cooperative metabolism, an early disturbance of microbiota–host communication by unhealthy dietary patterns may also affect the beneficial shield offered by commensals, thereby contributing to lifelong increased susceptibility to metabolic-related diseases.

## Figures and Tables

**Table 1:** Animal data and biochemical parameters.

	CD Group	HSD Group	CAFD Group	*p* Value
	(*n* = 9)	(*n* = 9)	(*n* = 9)	
BW_i (g)	114 (8)	112 (9)	116 (5)	0.59
BW_f (g)	438 (27)	410 (46)	457 (43)	0.07
BW gain (%)	284 (35)	269 (47)	293 (27)	0.49
Total WAT (g)	18 (4) ^a^	26 (10)	39 (7) ^a^	<0.0005
pgWAT (g)	8.8 (2) ^a^	13.0 (5)	18.8 (4) ^a^	<0.0005
rWAT (g)	4.1 (1) ^a^	6.1 (2) ^c^	10.0 (2) ^a c^	<0.0005
Total WAT (%)	4.1 (0.8) ^a^	6.3 (1.7)	8.5 (1.0) ^a^	<0.0005
Glucose (mmol/L)	6.8 (1.4) ^a b^	10.8 (1.9) ^b^	11.5 (2.7) ^a^	<0.0005
TG (mmol/L)	1.36 (0.4) ^a^	2.03 (0.5)	2.72 (0.8) ^a^	<0.0005
Total cholesterol (mmol/L)	1.65 (0.2) ^a b^	2.24 (0.4) ^b^	2.59 (0.5) ^a^	<0.0005
HDL-c (mmol/L)	0.53 (0.2)	0.42 (0.1)	0.40 (0.1)	0.28
LDL-c (mmol/L)	0.50 (0.3)	0.82 (0.3)	0.89 (0.4)	0.05
Urea (mmol/L)	11.3 (5) ^a^	15.8 (4)	19.1 (6) ^a^	0.01
Creatinine (μmol/L)	40 (12) ^a b^	73 (21) ^b^	75 (26) ^a^	<0.005
TG/HDL-c ratio	2.92 (1.7) ^a^	5.08 (2.2)	7.48 (3.7) ^a^	<0.0005
UCR	0.31 × 10^−3^	0.23 × 10^−3^	0.28 × 10^−3^	0.87

Abbreviations: BW_f, final body weight; BW_i, initial body weight; CD, chow diet; CAFD, cafeteria diet; HSD, high-sugar diet; pgWAT, perigonadal white adipose tissue; rWAT, retroperitoneal adipose tissue; TG, triglyceride; UCR, urea to creatinine ratio. Conversion factors: To convert glucose to mg/dL, divide by 0.0555; triglycerides to mg/dL, divide by 0.0113; cholesterol to ml/dL, divide by 0.0295; urea to ml/dL divide by 0.3571; creatinine to mg/dL divide by 88.42. Values are mean (SD). *p* values reported for Kruskal-Wallis statistic. Multiple comparisons with Dunn test using Šidák correction within each variable. Common letters in superscript indicate statistically significant differences between the associated groups: ^a^ CD vs. CAFD; ^b^ CD vs. HSD; ^c^ HSD vs. CAFD.

**Table 2:** Summary of 16S rRNA sequence reads analysis from fecal samples of all groups.

	No. of Paired-End Sequences	No. of Sequences after Demultiplexing and Length-Filtering	No. of Sequences after Pre-Filtering by Similarity to Pre-Existing OTUs	No. of OTUs Using Identity Cutoff ≥ 97%	% of Sequences Not Assigned to Any Taxon	% of Sequences Corresponding to Uncultured Species
all animals	9.86 × 10^6^	6,781,901	6,634,094	68,269	0.25	88.6
CD group			av. 323,983 (min. 272,286–max. 399,253)	37,892	0.6	93.3
HSD group			av. 279,801 (min. 241,989–max. 333,040)	49,010	0.15	89.3
CAFD group			av. 277,330 (min. 248,466–max. 300,271)	49,991	0.15	84.8

Abbreviations: CD, chow diet; CAFD, cafeteria diet; HSD, high-sugar diet; OTU, operational taxonomic unit.

## Data Availability

Data generated or analyzed during this study are included in this article and its Supplementary Information and sequencing results are available through ENA-EBI under project identifier PRJEB23897 (sample accession numbers ERS2045493 to ERS2045515). Data in Brief or files are available from the corresponding author on reasonable request.

## Supplementary Materials

The following are available online at https://www.mdpi.com/article/10.3390/nu13093236/s1, Figure S1: Procedures for 16S rRNA gene sequencing and analysis, Table S1: Composition of the intervention diets, Table S2: Sequences statistics after pair-joining, demultiplexing length-filtering and 16rRNA pre-filtering, discriminated by sample, Table S3: Gut microbial community by group.

## References

[1] Conlon M.A., Bird A.R. (2015). The Impact of Diet and Lifestyle on Gut Microbiota and Human Health. Nutrients.

[2] Dominguez-Bello M.G., Godoy-Vitorino F., Knight R., Blaser M.J. (2019). Role of the microbiome in human development. Gut.

[3] Rampelli S., Guenther K., Turroni S., Wolters M., Veidebaum T., Kourides Y., Molnár D., Lissner L., Benitez-Paez A., Sanz Y. (2018). Pre-obese children’s dysbiotic gut microbiome and unhealthy diets may predict the development of obesity. Commun. Biol..

[4] Jumpertz R., Le D.S., Turnbaugh P.J., Trinidad C., Bogardus C., Gordon J.I., Krakoff J. (2011). Energy-balance studies reveal associations between gut microbes, caloric load, and nutrient absorption in humans. Am. J. Clin. Nutr..

[5] Turnbaugh P.J., Ley R.E., Mahowald M.A., Magrini V., Mardis E.R., Gordon J.I. (2006). An obesity-associated gut microbiome with increased capacity for energy harvest. Nature.

[6] Cani P.D., Everard A. (2016). Talking microbes: When gut bacteria interact with diet and host organs. Mol. Nutr. Food Res..

[7] Bäckhed F., Ding H., Wang T., Hooper L.V., Koh G.Y., Nagy A., Semenkovich C.F., Gordon J.I. (2004). The gut microbiota as an environmental factor that regulates fat storage. Proc. Natl. Acad. Sci. USA.

[8] Knudsen K.E.B., Lærke H.N., Hedemann M.S., Nielsen T.S., Ingerslev A.K., Nielsen D.S.G., Theil P.K., Purup S., Hald S., Schioldan A.G. (2018). Impact of Diet-Modulated Butyrate Production on Intestinal Barrier Function and Inflammation. Nutrients.

[9] Cox L., Blaser M.J. (2015). Antibiotics in early life and obesity. Nat. Rev. Endocrinol..

[10] De Filippo C., Cavalieri D., Di Paola M., Ramazzotti M., Poullet J.B., Massart S., Collini S., Pieraccini G., Lionetti P. (2010). Impact of diet in shaping gut microbiota revealed by a comparative study in children from Europe and rural Africa. Proc. Natl. Acad. Sci. USA.

[11] Nobs S.P., Zmora N., Elinav E. (2020). Nutrition Regulates Innate Immunity in Health and Disease. Annu. Rev. Nutr..

[12] Karvonen A.M., Sordillo J.E., Gold D.R., Bacharier L.B., O’Connor G.T., Zeiger R., Beigelman A., Weiss S.T., Litonjua A.A. (2018). Gut microbiota and overweight in 3-year old children. Int. J. Obes..

[13] Leigh S.-J., Morris M.J. (2020). Diet, inflammation and the gut microbiome: Mechanisms for obesity-associated cognitive impairment. Biochim. Biophys. Acta (BBA)-Mol. Basis Dis..

[14] Noble E.E., Olson C.A., Davis E., Tsan L., Chen Y.-W., Schade R., Liu C., Suarez A., Jones R.B., de La Serre C. (2021). Gut microbial taxa elevated by dietary sugar disrupt memory function. Transl. Psychiatry.

[15] Ramos-Romero S., Hereu M., Atienza L., Casas J., Jáuregui O., Amézqueta S., Dasilva G., Medina I., Nogués M.R., Romeu M. (2018). Mechanistically different effects of fat and sugar on insulin resistance, hypertension, and gut microbiota in rats. Am. J. Physiol. Endocrinol. Metab..

[16] Nagpal R., Wang S., Woods L.C.S., Seshie O., Chung S.T., Shively C.A., Register T.C., Craft S., McClain D.A., Yadav H. (2018). Comparative Microbiome Signatures and Short-Chain Fatty Acids in Mouse, Rat, Non-human Primate, and Human Feces. Front. Microbiol..

[17] Kundu P., Lee H.U., Garcia-Perez I., Tay E.X.Y., Kim H., Faylon L.E., Martin K.A., Purbojati R., Drautz-Moses D.I., Ghosh S. (2019). Neurogenesis and prolongevity signaling in young germ-free mice transplanted with the gut microbiota of old mice. Sci. Transl. Med..

[18] Lozupone C.A., Stombaugh J., Gordon J.I., Jansson J.K., Knight R. (2012). Diversity, stability and resilience of the human gut microbiota. Nature.

[19] Bortolin R.C., Vargas A.R., Gasparotto J., Chaves P.R., Schnorr C.E., Martinello K.B., Silveira A.K., Rabelo T.K., Gelain D.P., Moreira J.C.F. (2018). A new animal diet based on human Western diet is a robust diet-induced obesity model: Comparison to high-fat and cafeteria diets in term of metabolic and gut microbiota disruption. Int. J. Obes..

[20] Fuke N., Nagata N., Suganuma H., Ota T. (2019). Regulation of Gut Microbiota and Metabolic Endotoxemia with Dietary Factors. Nutrients.

[21] Carmody R.N., Gerber G.K., Luevano J.M., Gatti D.M., Somes L., Svenson K.L., Turnbaugh P.J. (2015). Diet Dominates Host Genotype in Shaping the Murine Gut Microbiota. Cell Host Microbe.

[22] Lozano I., Van Der Werf R., Bietiger W., Seyfritz E., Peronet C., Pinget M., Jeandidier N., Maillard E., Marchioni E., Sigrist S. (2016). High-fructose and high-fat diet-induced disorders in rats: Impact on diabetes risk, hepatic and vascular complications. Nutr. Metab..

[23] Kübeck R., Bonet-Ripoll C., Hoffmann C., Walker A., Müller V.M., Schüppel V.L., Lagkouvardos I., Scholz B., Engel K.-H., Daniel H. (2016). Dietary fat and gut microbiota interactions determine diet-induced obesity in mice. Mol. Metab..

[24] Lee S.M., Kim N., Yoon H., Nam R.H., Lee D.H. (2018). Microbial Changes and Host Response in F344 Rat Colon Depending on Sex and Age Following a High-Fat Diet. Front. Microbiol..

[25] Crawford M., Whisner C., Al-Nakkash L., Sweazea K.L. (2019). Six-Week High-Fat Diet Alters the Gut Microbiome and Promotes Cecal Inflammation, Endotoxin Production, and Simple Steatosis without Obesity in Male Rats. Lipids.

[26] Caesar R., Tremaroli V., Kovatcheva-Datchary P., Cani P.D., Bäckhed F. (2015). Crosstalk between Gut Microbiota and Dietary Lipids Aggravates WAT Inflammation through TLR Signaling. Cell Metab..

[27] Hariri N., Gougeon R., Thibault L. (2010). A highly saturated fat-rich diet is more obesogenic than diets with lower saturated fat content. Nutr. Res..

[28] LeBlanc J., Labrie A. (1997). A possible role for palatability of the food in diet-induced thermogenesis. Int. J. Obes..

[29] Dowd S.E., Callaway T.R., Wolcott R.D., Sun Y., McKeehan T., Hagevoort R.G., Edrington T.S. (2008). Evaluation of the bacterial diversity in the feces of cattle using 16S rDNA bacterial tag-encoded FLX amplicon pyrosequencing (bTEFAP). BMC Microbiol..

[30] Chiodini R.J., Dowd S., Chamberlin W.M., Galandiuk S., Davis B., Glassing A. (2015). Microbial Population Differentials between Mucosal and Submucosal Intestinal Tissues in Advanced Crohn’s Disease of the Ileum. PLoS ONE.

[31] Kuczynski J., Stombaugh J., Walters W.A., González A., Caporaso J.G., Knight R. (2011). Using QIIME to Analyze 16S rRNA Gene Sequences from Microbial Communities. Curr. Protoc. Bioinform..

[32] Shen W., Le S., Li Y., Hu F. (2016). SeqKit: A Cross-Platform and Ultrafast Toolkit for FASTA/Q File Manipulation. PLoS ONE.

[33] Vázquez-Baeza Y., Pirrung M., Gonzalez A., Knight R. (2013). EMPeror: A tool for visualizing high-throughput microbial community data. GigaScience.

[34] Segata N., Izard J., Waldron L., Gevers D., Miropolsky L., Garrett W.S., Huttenhower C. (2011). Metagenomic biomarker discovery and explanation. Genome Biol..

[35] Morgan X.C., Tickle T., Sokol H., Gevers D., Devaney K.L., Ward D.V., Reyes J., Shah S., Leleiko N., Snapper S.B. (2012). Dysfunction of the intestinal microbiome in inflammatory bowel disease and treatment. Genome Biol..

[36] Wu G.D., Chen J., Hoffmann C., Bittinger K., Chen Y.Y., Keilbaugh S.A., Bewtra M., Knights D., Walters W.A., Knight R. (2011). Linking Long-Term Dietary Patterns with Gut Microbial Enterotypes. Science.

[37] Sonnenburg E.D., Smits S.A., Tikhonov M., Higginbottom S.K., Wingreen N.S., Sonnenburg J.L. (2016). Diet-induced extinctions in the gut microbiota compound over generations. Nat. Cell Biol..

[38] Del Bas J.M., Guirro M., Boqué N., Cereto A., Ras R., Crescenti A., Caimari A., Canela N., Arola L. (2017). Alterations in gut microbiota associated with a cafeteria diet and the physiological consequences in the host. Int. J. Obes..

[39] Noble E.E., Hsu T.M., Jones R.B., Fodor A., Goran M., Kanoski S. (2017). Early-Life Sugar Consumption Affects the Rat Microbiome Independently of Obesity. J. Nutr..

[40] Castro H., Pomar C.A., Pico C., Sánchez J., Palou A. (2014). Cafeteria diet overfeeding in young male rats impairs the adaptive response to fed/fasted conditions and increases adiposity independent of body weight. Int. J. Obes..

[41] Sampey B., Vanhoose A.M., Winfield H.M., Freemerman A.J., Muehlbauer M.J., Fueger P.T., Newgard C.B., Makowski L. (2011). Cafeteria Diet Is a Robust Model of Human Metabolic Syndrome With Liver and Adipose Inflammation: Comparison to High-Fat Diet. Obesity.

[42] Johnson A.R., Wilkerson M.D., Sampey B., Troester M.A., Hayes D.N., Makowski L. (2016). Cafeteria diet-induced obesity causes oxidative damage in white adipose. Biochem. Biophys. Res. Commun..

[43] Viraragavan A., Willmer T., Patel O., Basson A., Johnson R., Pheiffer C. (2021). Cafeteria diet induces global and Slc27a3-specific hypomethylation in male Wistar rats. Adipocyte.

[44] Lalanza J.F., Snoeren E.M. (2021). The cafeteria diet: A standardized protocol and its effects on behavior. Neurosci. Biobehav. Rev..

[45] Cluny N.L., Eller L.K., Keenan C.M., Reimer R.A., Sharkey K.A. (2015). Interactive effects of oligofructose and obesity predisposition on gut hormones and microbiota in diet-induced obese rats: Prebiotic Fiber and Obesity Predisposition. Obesity.

[46] St-Onge M.-P., Keller K.L., Heymsfield S.B. (2003). Changes in childhood food consumption patterns: A cause for concern in light of increasing body weights. Am. J. Clin. Nutr..

[47] Luque V., Escribano J., Closa-Monasterolo R., Zaragoza-Jordana M., Ferré N., Grote V., Koletzko B., Totzauer M., Verduci E., ReDionigi A. (2018). Unhealthy Dietary Patterns Established in Infancy Track to Mid-Childhood: The EU Childhood Obesity Project. J. Nutr..

[48] Mazarello Paes V., Hesketh K., O’Malley C., Moore H., Summerbell C., Griffin S., van Sluijs E.M., Ong K.K., Lakshman R. (2015). Determinants of sugar-sweetened beverage consumption in young children: A systematic review. Obes. Rev..

[49] Geserick M., Vogel M., Gausche R., Lipek T., Spielau U., Keller E., Pfäffle R., Kiess W., Körner A. (2018). Acceleration of BMI in Early Childhood and Risk of Sustained Obesity. N. Engl. J. Med..

[50] Chen G.-C., Huang C.-Y., Chang M.-Y., Chen C.-H., Chen S.-W., Huang C.-J., Chao P.-M. (2011). Two unhealthy dietary habits featuring a high fat content and a sucrose-containing beverage intake, alone or in combination, on inducing metabolic syndrome in Wistar rats and C57BL/6J mice. Metabolism.

[51] Ferreira A., Castro J.P., Andrade J.P., Madeira M.D., Cardoso A. (2018). Cafeteria-diet effects on cognitive functions, anxiety, fear response and neurogenesis in the juvenile rat. Neurobiol. Learn. Mem..

[52] Camerotto C., Cupisti A., D’Alessandro C., Muzio F., Gallieni M. (2019). Dietary Fiber and Gut Microbiota in Renal Diets. Nutrients.

[53] Koleva P.T., Bridgman S.L., Kozyrskyj A.L. (2015). The Infant Gut Microbiome: Evidence for Obesity Risk and Dietary Intervention. Nutrients.

[54] Everard A., Belzer C., Geurts L., Ouwerkerk J.P., Druart C., Bindels L.B., Guiot Y., Derrien M., Muccioli G.G., Delzenne N.M. (2013). Cross-talk between Akkermansia muciniphila and intestinal epithelium controls diet-induced obesity. Proc. Natl. Acad. Sci. USA.

[55] Villamil S.I., Huerlimann R., Morianos C., Sarnyai Z., Maes G.E. (2018). Adverse effect of early-life high-fat/high-carbohydrate (“Western”) diet on bacterial community in the distal bowel of mice. Nutr. Res..

[56] Devkota S., Wang Y., Musch M.W., Leone V., Fehlner-Peach H., Nadimpalli A., Antonopoulos D.A., Jabri B., Chang E.B. (2012). Dietary-fat-induced taurocholic acid promotes pathobiont expansion and colitis in Il10−/− mice. Nat. Cell Biol..

[57] Shin N.-R., Whon T.W., Bae J.-W. (2015). Proteobacteria: Microbial signature of dysbiosis in gut microbiota. Trends Biotechnol..

[58] An J., Zhao X., Wang Y., Noriega J., Gewirtz A.T., Zou J. (2021). Western-style diet impedes colonization and clearance of Citrobacter rodentium. PLOS Pathog..

[59] Desai M.S., Seekatz A.M., Koropatkin N.M., Kamada N., Hickey C.A., Wolter M., Pudlo N.A., Kitamoto S., Terrapon N., Muller A. (2016). A Dietary Fiber-Deprived Gut Microbiota Degrades the Colonic Mucus Barrier and Enhances Pathogen Susceptibility. Cell.

[60] Chassaing B., Koren O., Goodrich J.K., Poole A.C., Srinivasan S., Ley R.E., Gewirtz A.T. (2015). Dietary emulsifiers impact the mouse gut microbiota promoting colitis and metabolic syndrome. Nature.

[61] Turnbaugh P.J., Hamady M., Yatsunenko T., Cantarel B.L., Duncan A., Ley R.E., Sogin M.L., Jones W.J., Roe B.A., Affourtit J.P. (2009). A core gut microbiome in obese and lean twins. Nature.

