# Supplementary material for: Gut Microbiome Composition and Metabolic Status Are Differently Affected by Early Exposure to Unhealthy Diets in a Rat Model
Source: Nutrients. 2021 Sep 17;13(9):3236. doi: 10.3390/nu13093236 (PMC8469890; doi:10.3390/nu13093236)
Supplement: Supplementary file 1 [file nutrients-13-03236-s001.zip › supplemental table 1 and 2.pdf]

Supplement 1.

Table S1. Composition of the intervention diets

At 4 weeks of age, rats (CD) were fed with standard chow diet (Mucedola, Milan, Italy) and water; high-sugar diet (HSD) group were fed with standard chow diet and a solution of 30% sucrose (Sigma-Aldrich Company Ltd., Madrid, Spain; 1.2 Kcal/mL); cafeteria diet (CAFD) group were fed with standard chow diet plus a mix of palatable foods (chocolate cake, biscuits, dog roll and a high-fat rat chow) and a solution of 15% sucrose (Sigma-Aldrich Company Ltd., Madrid, Spain; 0.6 Kcal/mL).

**Compositions**	**CD** (chow diet)	**HSD** (chow diet + sucrose solution)	**CAFD** (mixed food + sucrose solution)
**Food energy content** (Kcal/g)	**3.9**	**3.9**	~ **4.5**
**Protein** (% Kcal)	**20**	**20**	**12**
**CHO** (% Kcal)	**68**	**68**	**43**
of which sucrose (% CHO)	--	--	~ 40
**Fat** (% Kcal)	**12**	**12**	**45**
saturated fat (% fat)	1.3	1.3	~ 38
**Drink energy content** (Kcal/mL)	**--**	**1.2**	**0.6**

Abbreviations: %, percentage; CD, chow diet; CHO, carbohydrates; HSD, high-sugar diet; CAFD, cafeteria diet

Table S2. Sequences statistics after pair-joining, demultiplexing length-filtering and 16rRNA pre-filtering, discriminated by sample

Sample	Number of reads	Length		Number of bases	Ns
		Mean	SD		
**CD1**	399253	289.8058	4.339368	117958213	0
**CD2**	337421	289.7531	4.405118	99788070	0
**CD3**	315032	289.7421	4.383879	92997067	0
**CD4**	295921	289.6844	4.35879	87420970	0
**CD5**	272286	289.5394	4.623917	80525836	0
**HSD1**	261747	289.6338	4.473185	77338307	0
**HSD2**	308018	289.7032	4.43724	91278805	0
**HSD3**	281392	289.6942	4.423543	83257535	0
**HSD4**	261540	289.7139	4.424959	77443709	0
**HSD5**	268744	289.8103	4.234542	79184005	0
**HSD6**	304374	289.8996	4.236857	90051233	0
**HSD7**	333040	289.8521	4.293295	98790581	0
**HSD8**	257364	289.6048	4.466156	75917576	0
**HSD9**	241989	289.8426	4.352357	71638075	0
**CAFD1**	284701	289.7622	4.337894	84081737	0
**CAFD2**	248834	289.7758	4.324024	73350375	0
**CAFD3**	279746	289.9536	4.122732	81910163	0
**CAFD4**	293319	289.6203	4.339388	86127580	0
**CAFD5**	292963	289.7252	4.329329	86163973	0
**CAFD6**	248466	289.7277	4.300956	73081491	0
**CAFD7**	262055	289.8382	4.222183	77221296	0
**CAFD8**	285618	291.4155	2.048997	91632693	0
**CAFD9**	300271	289.6989	4.331058	88398154	0

Table S3. Gut microbial community by group

**Taxon**	**CD (av. %)**	**HSD (av. %)**	**CAFD (av.%)**
Archaea[(1)]	0.0020	0.0003	0
Firmicutes	74.4	65.4	49.8
(class Clostridia)	(93.7)[(5)]	(92)[(5)]	(83)[(5)]
Bacteroidetes[(2)]	21	30.7	40.5
Verrucomicrobia (99.99% *Akkermansia* genus)	2.05	1.4	4.71
Tenericutes/ class Mollicutes	1.7	0.8	0.5
Proteobacteria	0.23	0.96	3.1
(class Gammaproteobacteria)	0.03001	0.10633	0.77423
(order Enterobacteriales)	0.00948	0.08693	0.31387
Saccharibacteria	0.229	0.132	0.067
order Gastranaerophilales	0.074	0.164	0.874
**class Mollicutes/order RF39[(1)]**	**1.282**	**0.708**	**0.255**
ord. Bacillales[(1)]	0.47	0.03061	0.20432
fam. Planococcaceae[(1)]	0.01487	0.00029	0.00063
genus *Lysinibacillus*[(1)]	0.00020	0	0
genus *Planomicrobium*[(1)]	0.00531	0	0.00004
(fam. Bacillaceae) *Bacillus aerius*[(2)]	0.00018	0	0.00004
Synergistaceae[(1)]	0.00067	0.00024	0.00011
unc. *Pyramidobacter* sp. [(1)]	0.00013	0	0
Thermanaerovibrio acidaminovorans[(1)]	0.00013	0	0
fam. Cyclobacteriaceae /genus *Algoriphagus*[(1)]	0.00018	0	0
fam. Desulfomicrobiaceae / genus *Desulfomicrobium*[(1)]	0.00051	0	0
fam. Syntrophorhabdaceae / genus *Syntrophorhabdus*[(1)]	0.00013	0	0
fam. Oceanospirillaceae / unc. species of *Marinobacterium*[(1)]	0.00018	0	0
fam. Spongiibacteraceae / genus *Zhongshania*[(1)]	0.00034	0	0
genus *Leptolyngbya*[(1)]	0.00019	0	0
genus “Lachnospiraceae NC2004”[(1)]	0.011	0.003	0.008
genus “Lachnospiraceae UCG-004”[(1)]	0.0289	0.0301	0.0258
genus C. *Cloacimonas*[(1)]	0.00011	0	0
**unc. *Prevotella*[(1)]**	**0.00094**	**0.00081**	**0.00145**
unc. *Arcobacter*[(1)]	0.00062	0	0
genus *Mogibacterium*[(1)]	0.00160	0.00076	0.00053
genus *Flavonifractor*[(1)]	0.00288	0.00246	0.00146
unc. species of *Pseudoflavonifractor*[(1)]	0.00037	0.00008	0.00048
genus *Megasphaera*[(1)]	0.00086	0.00050	0.00028
**genus *Parvibacter*[(1)]**	**0.00231**	**0.00046**	**0.00108**
**genus *Senegalimassilia*[(1)]**	**0.01144**	**0.00393**	**0.00225**
Thermanaerovibrio acidaminovorans[(1)]	0.00013	0	0
unc. *Pyramidobacter*[(1)]	0.00013	0	0
fam. Desulfovibrionaceae / genus *Bilophila*	0.0182	0.0619	0.3228
fam. Peptococcaceae[(2)]	0.61250	0.33287	0.30818
genus *Aerococcus*[(2)]	0.00024	0.00004	0.00011
genus *Subdoligranulum*[(2)]	0.00077	0.00041	0.00039
Thiotricaceae / unc. *Thiothrix*[(2)]	0.00018	0.00032	0.00012
genus “*Selenomonas* 1”[(2)]	0.00130	0.00206	0.00160
unc. *Nistropira*[(3)]	0.00054	0.00016	0
unc. *Parvibaculum*[(3)]	0.00021	0.00027	0
unc. Halothiobacillaceae[(3)]	0.00023	0.00033	0.00004
unc. *Actinobacillus*[(3)]	0.00024	0.00004	0

genus “Succinivibrionaceae UCG_001”(3)	0.00017	0.00016	0
Pseudobutyrivibrio(3)	0.00067	0.00054	0
unc. “Lachnospiraceae UCG-001”(3)	0.42251	0.70699	0.31723
unc. *Caproiciproducens*(3)	0.00032	0.00008	0
unc. “Ruminococcaceae UCG-007”(3)	0.00187	0.00084	0.00055
Pseudoramibacter alactolyticus(3)	0.00012	0.00007	0
unc. *Acidaminococcus*(3)	0.00017	0.00021	0
genus “*Prevotella* 2” (3)	0.00011	0.00028	0.00051
genus “Ruminococcaceae NK4A214” (3) (4)	0.43109	0.23470	0.29703
genus *Rhizobium*(3) (4)	0	0.00026	0.00207
**genus *Oscillibacter***(4)	1.14851	0.85943	0.81998
genus *Geobacillus*(4)	0.00006	0.00063	0
Chloroflexi/order S085 unc. genus(4)	0	0.00043	0
**genus *Barnesiella***(4)	0.07500	0.15109	0.05021
unc. “Lachnospiraceae UCG-010”(4)	0.00451	0.00282	0.00449

Index (1) marks taxa that were found having LDA discriminant capacity between chow diet (CD) and the 2 unhealthy dietary treatments; (2) discriminant between CD and HSD; (3) discriminant between CD and CAFD; and (4) discriminant between HSD and CAFD. (5) expressed as percentage of the Firmicutes. unc. = uncultured
