# Supplementary figures and images for: Gut Microbiome Composition and Metabolic Status Are Differently Affected by Early Exposure to Unhealthy Diets in a Rat Model
Source: Nutrients. 2021 Sep 17;13(9):3236. doi: 10.3390/nu13093236 (PMC8469890; doi:10.3390/nu13093236)

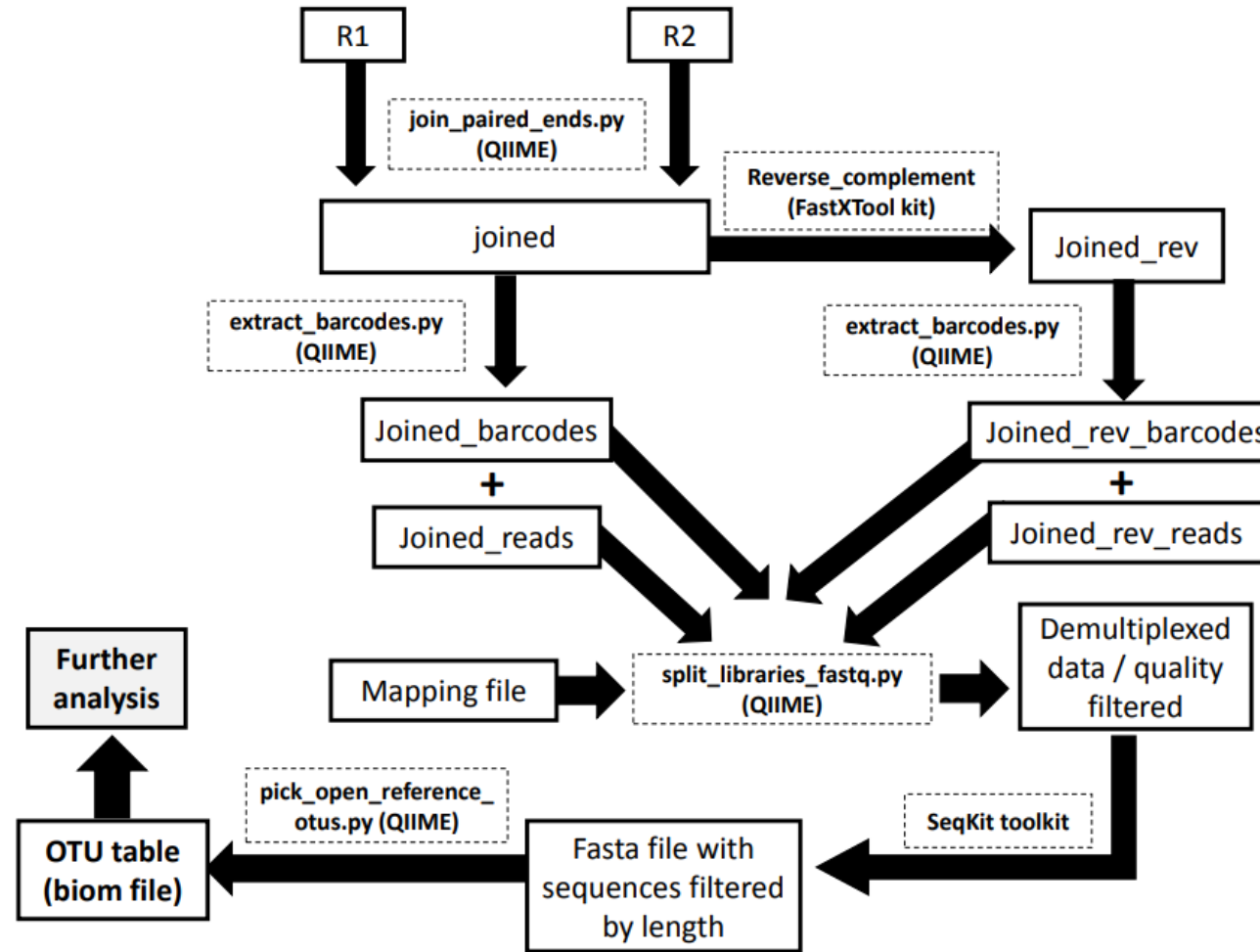

**Figure S1** – Procedures for 16S rRNA gene sequencing and analysis

Supplement: Supplementary file 1 [file nutrients-13-03236-s001.zip › supplemental_Figure S1.pdf]
